# The birth of the electric machines: a commentary on Faraday (1832) ‘Experimental researches in electricity’

**DOI:** 10.1098/rsta.2014.0208

**Published:** 2015-04-13

**Authors:** Jim Al-Khalili

**Affiliations:** Department of Physics, University of Surrey, Guildford, Surrey, UK

**Keywords:** electromagnetism, induction, dynamo, electric motor

## Abstract

The history of science is filled with examples of key discoveries and breakthroughs that have been published as landmark texts or journal papers, and to which one can trace the origins of whole disciplines. Such paradigm-shifting publications include Copernicus' De revolutionibus orbium coelestium (1543), Isaac Newton's Philosophiæ Naturalis Principia Mathematica (1687) and Albert Einstein's papers on relativity (1905 and 1915). Michael Faraday's 1832 paper on electromagnetic induction sits proudly among these works and in a sense can be regarded as having an almost immediate effect in transforming our world in a very real sense more than any of the others listed. Here we review the status of the subject—the relationship between magnetism and electricity both before and after Faraday's paper and delve into the details of the key experiments he carried out at the Royal Institution outlining clearly how he discovered the process of electromagnetic induction, whereby an electric current could be induced to flow through a conductor that experiences a changing magnetic field. His ideas would not only enable Maxwell's later development of his theory of classical electromagnetism, but would directly lead to the development of the electric dynamo and electric motor, two technological advances that are the very foundations of the modern world. This commentary was written to celebrate the 350th anniversary of the journal *Philosophical Transactions of the Royal Society*.

## 1. Electromagnetism before Faraday

The early nineteenth century was an exciting time for experimental physics. It was also a time of great confusion about the nature of electricity. The work of two Italians, Luigi Galvani and Alessandro Volta on the characteristics of bioelectricity had led Volta to invent the battery in 1799. His ‘voltaic pile’ suddenly gave natural philosophers (the term ‘scientists’ was not coined until 1834^1^) a reliable and far more useful source of electricity than the Leyden jars or ever-more sophisticated electrostatic machines and turned the subject from an intellectual curiosity into a proper science. In a very really sense, it *galvanized* science, which is of course the origin of that everyday word.

In particular, it was the confusing relationship between electricity and magnetism that so fascinated many scientists. Indeed, it was argued by some that there was no connection at all between the two phenomena, although it had been known since the mid-eighteenth century that, for example, thunderbolts created certain magnetic effects.

Then, in 1820, the Danish scientist Hans Christian Ørsted carried out an experiment for which he is credited with the discovery of electromagnetism. On 21 April 1820, he observed, while preparing for a lecture, that when he ran an electric current through a wire, a nearby compass needle was temporarily deflected from its stable position of pointing towards the magnetic north. This happened at the moment the current from a battery was switched on, and then again when it was switched off, thus confirming a direct relationship between electricity and magnetism, namely that the change in an electric current (from none to flowing, and vice versa) produced a temporary magnetic effect in its vicinity.

An account of Ørsted's discovery comes in a letter his colleague Christopher Hansteen wrote to Michael Faraday many years later:

Ørsted had tried to place the wire of his galvanic battery perpendicular (at right angles) over the magnetical needle, but remarked no sensible motion. Once, after the end of his lecture as he had used a strong galvanic battery to other experiments, he said: ‘let us now once, as the battery is in activity, try to place the wire parallel with the needle’. As this was made he was quite struck with perplexity by seeing the needle making a great oscillation (almost at right angles with the magnetic meridian). Then he said: ‘let us now invert the direction of the current’, and the needle deviated in the contrary direction.^2^

Ørsted's original interpretation was that the magnetic effects produced by the current through the wire radiated outwards in the same way that heat or light does. But after further experimentation he showed that in fact the produced magnetic field circled around the wire (although of course no one was yet thinking in terms of fields).

Within months of Ørsted's discovery, the French physicist and mathematician André-Marie Ampère had shown that two current carrying wires placed in parallel close to each other each generated magnetic lines of force that caused the wires to be attracted or repelled from each other depending on whether the currents were flowing in the same or in opposite directions. Ampère would go on to help found the field of classical electromagnetism and has the SI unit of electric current named after him.

Ampère and Ørsted had shown that, somehow, electricity could be converted into magnetism, but they and others had failed to do the reverse: to create electricity *from* magnetism.

An equally famous (at the time, though less so today) French physicist by the name of François Arago then carried out an experiment that completely baffled most scientists at the time and served as one of the main motivations for Faraday's great work. In 1824, Arago demonstrated that a spinning copper disc caused a magnetic needle suspended above it to rotate. This result was remarkable for two reasons. Firstly, there was no external electric current being applied to the copper disc and, secondly, although copper is a conductor, it is not magnetic. Yet here was a magnetic field seemingly being produced just by the rotation of this disc that was influencing the compass needle. It would take the brilliance and inventiveness of Michael Faraday to describe what was going on and, in a series of carefully and clearly described experiments between August and November 1831, he would change the face of science in ways that have an impact on all our lives to this day.

## 2. Early life

Michael Faraday was born in 1791 in Newington Butts, now in South London, but then no more than a village in rural Surrey. He was the son of a blacksmith who had moved down from Cumbria in northwest England just before Michael was born. His family were not well off and Faraday received a typical working class education, which he compensated for by reading all the books he could lay his hands on, a passion that he fed by becoming an apprentice to a bookbinder and bookseller at the age of 14. His growing fascination with science led, in 1812, to an event that would change his life and the course of human history: a kindly customer of the bookshop offered the young Faraday tickets to attended a series of lectures given by the great Humphry Davy at the Royal Institution, which had been founded a few years earlier. When he presented Davy with the copious notes he had taken during the lectures the great man was so impressed that he took him on as his laboratory assistant.^3^ The following year, Faraday travelled with Davy around Europe where he would have seen and heard many of the leading thinkers of the age [4]. When, in 1820, he heard about Ørsted's experiment he determined to conduct his own investigations into the nature of electromagnetism.

What Faraday lacked in formal scientific training, particularly in mathematics, he made up for by his exceptional talent as an experimentalist. And although he was initially distrustful of mathematics, regarding it as obstructing rather than helping our understanding of the workings of Nature, he would, later in life, change his view in the light of the work of James Clerk Maxwell. In fact, Maxwell himself regarded Faraday as being an excellent theoretician and claimed this was the reason he was able to put Faraday's theories into the language of analytical mathematics. It is important to point out that there are in fact three concepts that are intricately linked in Faraday's research: an electric current, a magnetic field and mechanical motion, and it is the interplay between these three that is the constant theme throughout his work. In September 1821, he built a device that can be regarded as the very first electric motor. He showed that a suspended wire hanging freely in a container of mercury with a permanent magnet in its centre would rotate around the magnet once an electric current was passed through it (with the conducting mercury completing the circuit).

With this beautifully simple set-up Faraday had demonstrated what he called ‘electromagnetic rotations’—he had used both electricity and magnetism to generate motion, a generalization of Ørsted's principle—and had discovered the principle behind the electric motor [5]. The idea being that the current through the wire created a magnetic field around it, which interacted with the field of the magnet, pushing the wire around in a circular motion all the while the current was flowing.

This early discovery so excited Faraday that he spent the next decade, on and off, trying to understand the physics behind electromagnetism. He described in his diary at the time a number of failed experiments in which he tried to demonstrate what he called ‘Electro Magnetic Induction’.^4^ But his investigations following the work of Ørsted, Ampère and Arago were put on temporary hold between 1825 and 1830 when he became preoccupied, on the instructions of Davy, with finding ways to improve the quality of optical glass used for lenses. Everything changed during the second half of 1831, a year which culminated in the paper we can now describe. Having generated continuous mechanical motion from magnetism and electricity (the electric motor), the scene was set to generate electricity from mechanical motion and magnetism (the electric dynamo, or generator)—a discovery that would transform our world.

Recall that Faraday's overriding motivation was to ‘Convert Magnetism into Electricity’ [6] and it was Arago's disc that was the launch pad for his beautiful experiments. What makes his work so wonderful to read today of course is the clarity and precision of his descriptions. William Henry Bragg, who would much later become Director of the Davy-Faraday Research Laboratory at the Royal Institution in 1923, wrote in the foreword of a book about Faraday celebrating the centenary of his discover:

Faraday was in the habit of describing each experiment, in full and careful detail, on the day on which it was made. Many of the entries discuss the consequences, which he would draw from what he had observed. In other cases they outline the proposed course of a research about to be undertaken. Thus the Diary is far more than a catalogue of results. The reader is able to follow advance, step by step, to final and fundamental conclusions. He sees the idea forming, its experimental realisation, and its employment as a foothold for the next advance. [7]

## 3. Experimental researches in electricity

The paper that is the focus of this article is the first, and most famous, of a series of thirty papers Faraday wrote and published in the Royal Society's *Philosophical Transactions* between 1832 and 1856 under the title of *Experimental Researches in Electricity*, each taking up where the previous left off. This first one was read to the Royal Society on 24 November 1831.^5^ However, it was around this time that the Royal Society introduced a new refereeing procedure for papers submitted for publication in its *Philosophical Transactions*, which was to delay the appearance of Faraday's paper in print by several months, much to his frustration [8].

On 14 January 1832, worried about being beaten to the post by the French, he wrote to the Secretary of the Royal Society urging him to try and speed up the publication process ‘or else these philosophers may get some of my facts in conversation, repeat them & publish in their own name before I am out’.^6^ Although the exact date of the appearance of the paper in print is unknown, the earliest evidence we have for it is 9 April 1832.^7^

The experiments described in the paper all showed, with increasing sophistication, that a current could be induced all the time there was relative motion between the conductor and the magnetic field. With hindsight and the appropriate language we use today, we say that a current is induced in a conductor when it is inside a changing magnetic field. For this to happen, it does not matter whether it is the conducting wire or the object producing the magnetic field (either a permanent magnet or another wire with an electric current flowing through it) that is actually moving.

The first experiment Faraday discusses in the paper demonstrates simple induction and is worth describing here. First, 26 feet of copper wire were wound round a wooden cylinder as a helix. Insulating the individual spires and preventing them touching each other by a thin interposed twine. Then the wire coil was covered by a layer of muslin (a thick cotton fabric, also called calico—the name commonly used at the time, including by Faraday). Next a second copper wire was wound on top of this. This process was repeated until he had 12 coils of wire, all insulated from each other. He then connected the free ends of all the even coils to make one continuous length and likewise with the odd coils. He now had two helices, the ends of one of which he connected to a galvanometer (a device invented a few years earlier to detect the presence of an electric current) and the other to a voltaic battery.

At first, Faraday saw no response from the galvanometer when switching on the battery, but by experimenting with longer coiled wires, different materials for the conducting wires and more powerful batteries he was finally able to induce a small reaction in the galvanometer needle, deflecting one way on switching the battery on and the other when disconnecting it. We now know this effect as electro-magnetic induction – in the sense that changes in the electric current in the first wire, and hence the magnetic field produced by it, was inducing a current to temporarily flow in the second wire.

He next found a far more effective way of changing the magnetic field: by moving two wires, one connected to a battery and the other to a galvanometer, towards or away from each other. The galvanometer needle reacted by vibrating one way then the other in step with the motion of the wires to and fro. But as soon as they were brought to rest so did the galvanometer needle, indicating no more current through the second wire, even though it continued to flow continuously through the first.

It is worth noting that at this point Faraday, in common with other researchers at the time, still didn't understand the nature of electricity itself. He refers to the electricity flowing through a wire due to a voltaic battery as *voltaic electricity* and the effect it having on the second wire as *volta-electric induction*. He distinguishes this from the electrical discharge from a Leyden jar as *electricity of tension* or *ordinary electricity*. It would not be until he built the first Faraday cage in 1836 that he began thinking of electricity as a force rather than a fluid.

He next moves on to a far more effective version of his first experiment in which he attempts to induce a current in a coil of wire due to switching on and off the current in another coil. This time he used an un-magnetized iron ring instead of the original wooden cylinder. He wound two coils of wire on opposite sides of the ring being very careful to insulate them from the ring itself and, by separating each loop of the wires from adjacent ones by insulating thread. Then he connected one coil to a battery and the other coil to a galvanometer. At the instance that the battery was switched on ‘the galvanometer was immediately affected, and to a degree far beyond what has been described’ ([9], §28) and it was again powerfully deflected when the battery was switched off. Clearly, a temporary current was being generated in the second wire every time he connected and disconnected the battery. One can almost sense the excitement as Faraday writes:

Upon using the power of one hundred pair of plates [to create as powerful a battery as possible from his voltaic pile] with this ring, the impulse at the galvanometer, when contact was completed or broken, was so great as to make the needle spin round rapidly four or five times before the air and terrestrial magnetism could reduce its motion to mere oscillations. ([9], §31)

**Figure 1.**
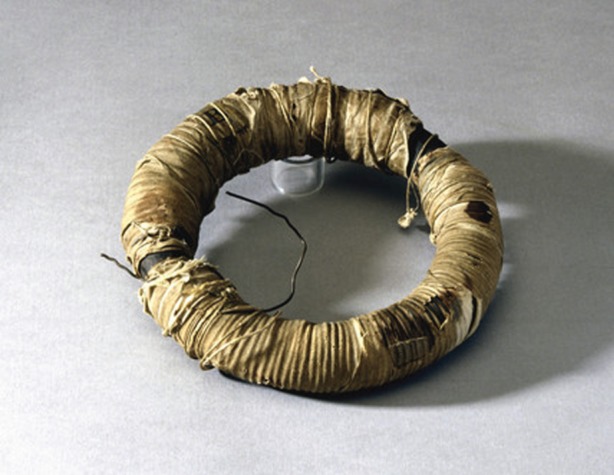
Faraday's induction ring (1831). Image courtesy of the Royal Society/Science and Society Picture Library.

Faraday's induction ring was, in effect, the very first electrical transformer. It survives to this day and is on display in the Royal Institution's museum (figure 1). There is no doubt that this remains one of the most important scientific objects the history of science.

Faraday then went on to note that by replacing the iron ring with a copper one the induced current was far weaker, and similar to when the coiled wires where not wound round anything at all. Clearly, the difference here is that the iron ring was helping generate a much stronger electromagnet, in a way that non-magnetic copper could not do.

The next step was an important one. Faraday realized that there had to be ‘some peculiar effect taking place during the formation of the magnet, and not by its mere virtual approximation, that the momentary induced current was excited’ ([9], §39). He carried out an experiment that is to this day familiar in any science classroom in the world. He replaced the wire helix connected to the battery, and which was generating a magnetic field, with a simple permanent bar magnet. He next took a hollow coil of wire the ends of which he connected to a galvanometer. By thrusting the magnet quickly into the coil and saw the galvanometer needle deflect. Reversing the process by pulling the magnet out caused the needle to deflect in the opposite direction. Then, by constantly moving the bar magnet in and out of the coil he could make the galvanometer needle vibrate from side to side in phase with the motion of the magnet.

Faraday went on to experiment with more powerful permanent magnets and electromagnets of different strengths, but the basic principle was the same. He states triumphantly that ‘the various experiments…prove, I think, most completely the production of electricity from ordinary magnetism’ ([9], §57). He decides to refer to ‘the agency thus exerted by ordinary magnets’ as *magneto-electric induction* to distinguish it from *volta-electric induction* produced by the field of a current carrying wire. As for the second wire, which is subjected to this induction, he describes it as being ‘in a peculiar state’ of resisting the formation of an electric current in it and refers to it as being in an *electro-tonic* state. But at this point he admits that he has yet to understand the properties of matter whilst retained in this state, particularly since he experiments with various different conducting materials, such as copper and silver, which are not themselves magnetic.

**Figure 2.**
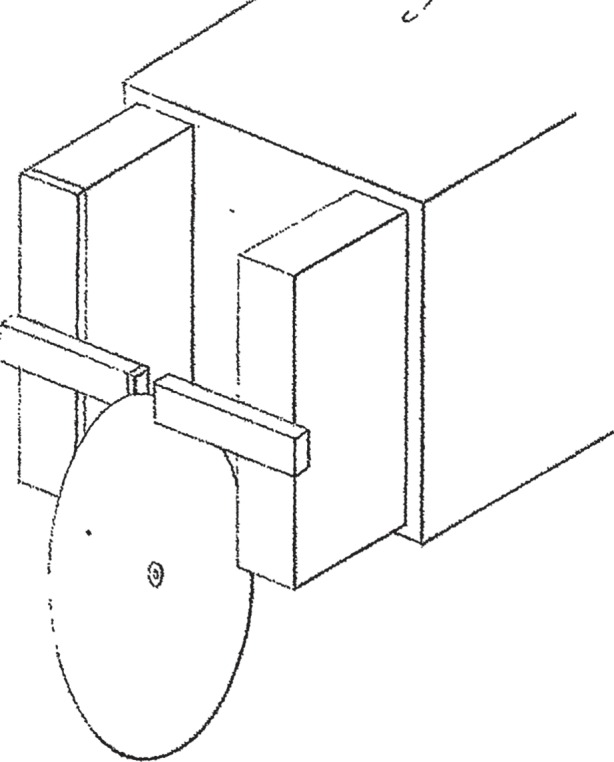
Faraday's spinning disc—generating a continuous electric current in a conducting disc as it spins between two poles of a powerful permanent magnet. This diagram is from Faraday's original paper [9]. Copyright The Royal Society.

Faraday realized he needed to find a way of producing a changing magnetic field and went on to design an improved version of Arago's disc experiment. He mounted a copper disc on a brass axes so that it could freely rotate between two poles of a permanent magnet. He then connected the disc to a galvanometer by attaching one wire to its centre and another touching its rim (as in figure 2).

Then, when the disc was rotated, the galvanometer registered a continuous current that clearly had to be travelling in a radial direction through the disc. Reversing the direction of spin of the disc caused the galvanometer needle to be deflected in the opposite direction implying a reversal in direction of the electric current.

Faraday famously noted that ‘Here therefore was demonstrated the production of a permanent current of electricity by ordinary magnets ([9], §90).’ His explanation of what was happening is beautifully clear: ‘If a terminated wire is moved so as to cut a magnetic curve, a power is called into action which tends to urge an electric current through it’ ([9], §256).^8^

With this experiment, Faraday was able to show how a magnetic field and continuous mechanical motion would produce a continuous electric current. He had invented the electric generator.

He then goes on to attach the two wires that connected to the galvanometer to different points on the rim of the spinning disc and realizes that the induced current is always at right angles to the motion of the disc and that in this case the flow of electricity is in a radial direction.

Faraday then makes an interesting and quite remarkable attempt at describing, at a more microscopic level, what might be going on inside metals carrying an induced electric current: ‘In the electro-tonic state the homogeneous particles of matter appear to have assumed a regular but forced electrical arrangement in the direction of the current… this forced state may be sufficient to make an elementary particle leave its companion, with which it is in a constrained condition, and associate with the neighbouring similar particle, in relation to which it is in a more natural condition’ ([9], §76). Note that he is adopting here Ampère's theory of electric current, but, viewed from the perspective of modern physics, one cannot help but marvel at his insight; for his description predates by over half a century Boltzmann's atomic theory and JJ Thomson's discovery of the electron, let alone an understanding of the nature of electricity as the flow of electrons.

Of course we can see how far away Faraday, and others, were at this time from understanding the true nature of electric current by the way he still refers to the different kinds of electricity. He defines five distinct types: Voltaic-Electricity (as produced by a battery), Common-Electricity (such as the discharge from a charged body like a Leyden jar), Magneto-Electricity (by which he means an induced current), Thermo-Electricity and Animal-Electricity (such as was known to be produced by some creatures such as the electric eel).

It should be mentioned at this point that the American scientist, Joseph Henry (1797–1878), whose life, starting from poor and humble beginnings, in many ways mirrored that of Michael Faraday, was also working (independently) on electro-magnetism on the other side of the Atlantic – although interest in the subject was certainly circulating across the Atlantic by the 1830s. Importantly, it is worth stating that Henry in fact beat Faraday to the discovery of inductance by a few months in 1831, but it was Faraday who published first and, despite the delays that so frustrated him, is therefore credited with the discovery.

## 4. Faraday's mistake

Today, every schoolboy and schoolgirl learns about Fleming's left- and right-hand rules. These useful visual mnemonics were developed by the English engineer, John Ambrose Fleming (1849–1945) in the late nineteenth century and give a simple way of working out the direction of motion in an electric motor (the left-hand rule) and the direction of current in a generator (the right-hand rule). For example, in the left hand rule, the index finger, middle finger and thumb can be held pointing in three mutually orthogonal directions to represent the magnetic Field (First finger), electric Current (seCond finger) and the thrust, or Motion, (thuMb). In reading Faraday's paper, one is struck by just how simple these mnemonics are and how useful they would have been had he known about them. In attempting to describe the direction of the induced current Faraday states: ‘The relation which holds between the magnetic pole, the moving wire or metal, and the direction of the current evolved, i.e. the law which governs the evolution of electricity by magneto-electric induction, is very simple, although rather difficult to express ([9], §114).’

Indeed, when experimenting with the two parallel wires, Faraday states: ‘As the wires approximated, the induced current was in the *contrary* direction to the inducing current. As the wires receded, the induced current was in the *same* direction as the inducing current ([9], §19).’ Then again a little later: ‘It was found in all cases that the induced current, produced by the first action of the inducing current, was in the contrary direction to the latter, but that the current produced by the cessation of the inducing current was in the same direction ([9], §26).’

**Figure 3.**
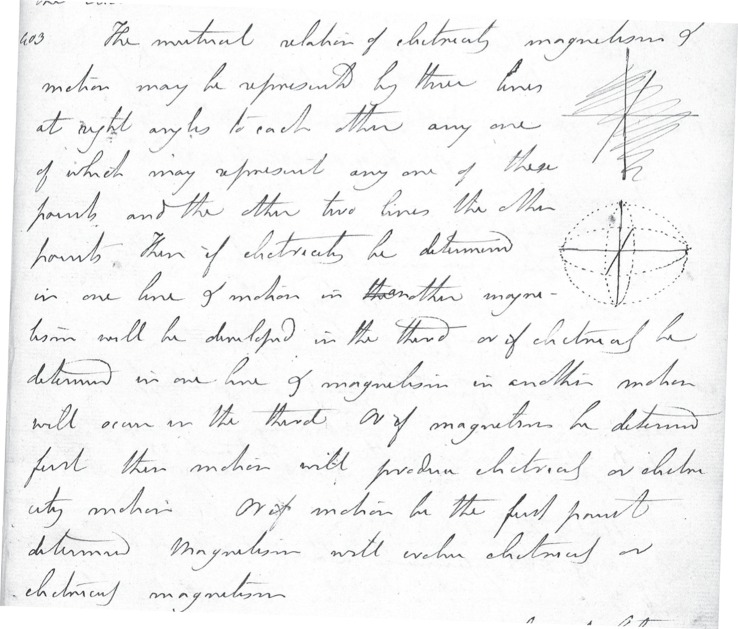
This is a page from Faradays' notebook written on 26 March 1832 (RI MS F/2/C, p. 147). It reads: ‘The mutual relation of electricity, magnetism and motion may be represented by three lines at right angles to each other, any one of which may represent any one of these points and the other two lines the other points. Then if electricity be determined in one line and motion in another, magnetism will be developed in the third; or if electricity be determined in one line and magnetism in another, motion will occur in the third. Or if magnetism be determined first then motion will produce electricity or electricity motion. Or if motion be the first point determined, Magnetism will evolve electricity or electricity magnetism’. Reproduced by courtesy of the Royal Institution of Great Britain.

But Faraday had got it the wrong way round [10]. Figure 3 shows an extract from his diary (his laboratory notebook) written on 26 March 1832, which was just a few days before his paper appeared in print and therefore too late for him to make any changes to it. We even see an interesting first attempt at drawing a diagram. The one below it depicts the correct mutual orthogonality of electricity, magnetism and motion and is regarded as one of the most significant drawings in his notebook.^9^

## 5. Impact of Faraday's discovery

There is no doubt that the experiments described in Faraday's paper not only laid the foundations for truly understanding the nature of electricity, but for its practical application in ways that would transform our world. Within months, many inventors became interested in these wondrous potential applications, and yet many of them did not understand, or even care, about the physics behind electromagnetic induction. Indeed, a true mathematical theory would not emerge until the work of James Clerk Maxwell in 1865.

The applications of Faraday's discoveries quickly became apparent as other scientists, engineers and inventors began to work on the construction of evermore-sophisticated electric generators that could be put to practical use [11]. For example, the French instrument maker, Hippolyte Pixii (1808–1835), built a crude electric generator as early as 1832, based directly on Faraday's ideas of induction. The device consisted of a hand operated spinning magnet above a coil with an iron core inside. A current pulse in the coil was produced each time one of the two poles of the magnet passed over it. However, what was being produced was an alternating (AC) current as the direction of the induced current changed with each half turn of the magnet. As there was no real use for AC currents at this time (its advantages would only become apparent later) a means had to be found to convert this into a direct (DC) current. A suggestion by Ampère and others led to the introduction of the commutator—a rotary switch that reverses the connection to the external circuit when the current reverses, giving a pulsing DC current instead of an AC one. Soon after Pixii's invention, others began to produce their own similar devices. Two London-based instrument makes to note were the American, Joseph Saxon, and the Englishman, Joseph Clarke. By the mid-1830s, such machines were producing a range of different effects of induced electric currents, from chemical decompositions to sparks, all by turning a handle that rotated a magnet.

The first important practical application of Faraday's discovery, however, was not the electric generator but the telegraph. Based on the ability to control a magnet at a distance, this invention allowed the possibility of long distance communication that would connect the world. And it was based on a very simple idea: the movement of a conducting coil over a magnet in one location induces a current that is transmitted to another location where it affects a galvanometer. The idea was implemented almost as soon as the world learnt of Faraday's work, particularly by Pavel Schilling, Carl Friedrich Gauss and Wilhelm Weber. Within a few years it was commercialized by Cooke and Wheatstone in Britain (1837) and by Morse and Vail in the USA (1838). A commercial, large-scale application of Faraday's discovery was made by the electroplaters of Birmingham as early as 1844. There, at least two companies made use of his method of extracting electricity from magnetism on a large scale [12].

The pace of invention then picked up in the 1850s as designs for evermore-powerful generators (known as ‘magneto-electric machines’) were developed in anticipation of the commercial applications of the electric light. But these early generators were incredibly cumbersome and of course required a source of power to produce the mechanical motion in the first place. The first experimental deployment of a magneto-electric machine powered by a steam engine took place in a British lighthouse. The device, which weighed 2 tons, was invented by the Englishman Frederick H. Holmes and first tested in the famous Bow Creek experimental lighthouse at Trinity Buoy Wharf on the river Thames in London in May 1857 under Faraday's supervision [13].^10^ It was then installed and used for the first time in the South Foreland Lighthouse on the Dover Cliffs the following year. As such, South Foreland became the first place in the world where electricity was generated for the practical provision of power. And, after 2000 years of using magnets for navigation, beginning with crude Chinese compasses using suspended lodestones, magnets were finally helping mariners in a different way: by generating powerful electric lights to guide them safely away from treacherous rocks.

By the mid-1860s, several scientists and inventors were developing practical designs for the dynamo-electric machine. These devices were using self-powering electromagnetic field coils instead of permanent magnets to enabled far greater power generation for the first time. As such, they led to the first major industrial uses of electricity and were the first generators capable of delivering sufficient power for industry.

After the discovery of the AC generator, now known as an alternator, the word dynamo became associated exclusively with the commutated DC electric generator. By the 1880s, the so-called ‘war of the currents’ was in full swing between those, such as Thomas Edison, who favoured DC current for power generation and those, led by George Westinghouse and Nikola Tesla, who believed that AC current was the way forward. The latter two would eventually and decisively win that bitter war. The development of AC power transmission, using transformers (whose origins lie in Faraday's simple induction ring) to transmit power at high voltage and with low loss, allowed central power stations to become economically practical.

Today, the alternator dominates large-scale power generation and relies on a fluid, usually steam, that acts as an intermediate energy carrier, to drive the turbines and generate electricity. In nuclear- and coal-powered power stations, the heat produced from nuclear fission and the chemical burning of carbon, respectively, is what is used to turn water into steam. In a sense, all power plants can be regarded crudely as giant kettles.

James Clark Maxwell (1831–1879) was born just a few months before Faraday conducted his famous experiments and became interested in the work on electromagnetic induction, and in particular what Faraday had begun referring to as the ‘lines of force’ to describe the influence of electric and magnetic fields. The young Maxwell would regularly attend Faraday's lectures at the Royal Institution and, as early as 1856, he published a paper entitled *On Faraday's Lines of Force* from which it is interesting to quote the following:

“I have attempted to bring before the mind, in a convenient and manageable form, those mathematical ideas which are necessary to the study of the phenomena of electricity. The methods are generally those suggested by the process of reasoning which are found in the researches of Faraday, and which, though they have been interpreted mathematically by Prof. Thomson and others, are very generally supposed to be of an indefinite and unmathematical character, when compared with those employed by the professed mathematicians. By the method which I adopt, I hope to render it evident that I am not attempting to establish any physical theory of a science in which I have hardly made a single experiment, and that the limit of my design is to shew how, by a strict application of the ideas and methods of Faraday, the connexion of the very different orders of phenomena which he has discovered may be clearly placed before the mathematical mind.”

A few years later, in 1861–62, Maxwell published a famous four-part paper entitled *On Physics Lines of Force*, which was followed, in 1865, by his greatest work [14], ‘A dynamical theory of the electromagnetic field’ [15] in which he unified electric and magnetic fields into one concept: a wave travelling through space at the speed of light and in which he laid out his famous equations for the first time (though not yet in the form of the four equations that bare his name and that are familiar to every physics student). This unification of light and electricity is regarded as one of the key advances in the history of science whereby mathematical flesh was added to Faraday's theories.

Maxwell's book [16] laid the foundations not just for the subsequent discovery of radio waves but for much of modern physics, including the work of Einstein on special relativity and the development of quantum theory, in the first decades of the twentieth century. This in turn led to the many wonderful advances that have shaped our modern electronic age, from television to computers to smart phones. As we look around us today we cannot fail to see the all-encompassing influence that Faraday's work has had on our lives—one that shows no sign of abating.

Throughout his life, Faraday was far more interested in understanding the underlying physical basis of electromagnetism and electromagnetic induction than many other scientists of his age who were rather more obsessed with putting his discoveries to practical use. Today, we still use Faraday as the best example of curiosity-driven scientific research carried out for its own sake.

## Footnotes

1 Many historians of science would regard the use of the term ‘scientist’ when referring to natural philosophers or chemists (including Faraday himself) as being anachronistic. However, henceforth in this paper we will nevertheless use this modern term, especially since there is just a two-year gap between Faraday's work and the invention of the word by Cambridge historian and philosopher, William Whewell in 1834.

2 Hansteen to Faraday, 30 December 1857, in [1, vol. 5, letter 3374].

3 For an account of Faraday's life see [2,3].

4 Faraday. 1825 *Diary* **1**, 279.

5 In fact, only the first part of the paper was read on 24 November. The remainder was read on two later occasions, on 8 and 15 December.

6 Faraday to Roget, 14 January 1832, in [1], vol. 2, letter 531.

7 Faraday to Hudson, 9 April 1832, in [1], vol. 2, letter 566.

8 Note that if series number not stated, then we denote the first paper, which is of course the subject of this article.

9 Frank James at the Royal Institution is currently preparing an electronic edition of Faraday's laboratory notebook covering his induction experiments.

10 Holmes's earliest machine, for direct current, incorporated 120 permanent magnets of horseshoe shape, each weighing 50 pounds, along with 160 coils; subsequent versions were somewhat smaller.

## Author profile

**Jim Al-Khalili** is a physicist, author and broadcaster based at the University of Surrey where he currently teaches and conducts research in quantum physics. He received his PhD in theoretical nuclear physics in 1989 and has published widely on the structure and reactions of exotic nuclei. He is active as a science communicator and has written a number of popular science books, between them translated into over twenty languages. His book, Pathfinders, on the history of medieval Arabic science was shortlisted for the Warwick Prize in 2013. His latest is Life on the Edge: the coming of age of quantum biology. He is a regular presenter of TV science documentaries, including the Bafta nominated Chemistry: A Volatile History and Shock and Awe: The Story of Electricity, both for the BBC. For the past three years he has presented the highly successful weekly BBC Radio 4 programme, The Life Scientific. In 2007, he won the Royal Society's Michael Faraday medal for science communication.
